# A Rare Case of Breast Metastatic Gastrointestinal Stromal Tumor

**DOI:** 10.7759/cureus.34164

**Published:** 2023-01-24

**Authors:** Woong Kee Baek, Pu Ni, Jennifer Zeng, Victoria Collins, Nebras Zeizafoun

**Affiliations:** 1 Department of Pathology, Mount Sinai Morningside - West, New York, USA; 2 Department of Pathology, Mount Sinai Hospital, New York, USA

**Keywords:** kit exon 17 mutation gist, kit exon 11 mutation gist, gist in immunosuppressed, breast metastasis of gist, metastatic gist, gastrointestinal stromal tumor (gist)

## Abstract

Gastrointestinal stromal tumor (GIST) is one of the most common spindle cell neoplasms of the alimentary system, and can arise anywhere along the gastrointestinal tract (GI). Its incidence rate is up to 22 cases per million, with a minor geographic variation. GIST is thought to originate from interstitial cell of Cajal, and its pathogenesis is related to molecular defects, such as KIT receptor tyrosine kinase or platelet-derived growth receptor alpha gene activation. While the majority of GISTs are known to show a benign disease course, metastases of high-grade forms to different organ systems have been seldom reported. We present a case with an unprecedented metastasis of GIST to the breast. The patient is a 62-year-old female with a history of the primary resection of GIST from the small intestine. Her disease course was initially complicated by multiple metastases, solely localized to the liver for which she had a living-donor liver transplant. The tumor harbored both KIT exon 11 and exon 17 mutation. Fourteen months post-transplant, the patient was found to have metastatic GIST on her breast biopsy. GIST metastasis to the breast is extremely rare. A consideration of this spindle cell neoplasm as a differential is recommended when clinical suspicion arises. The pathophysiology, current diagnostic tool, grading system, and treatment of this tumor are discussed.

## Introduction

Gastrointestinal stromal tumor (GIST) is one of the common neoplasms of mesenchymal cell origin, arising from the gastrointestinal (GI) tract [1]. While GIST is widely believed to arise from interstitial cell of Cajal, many studies have pointed molecular defects or syndromic diseases, as being responsible for its pathogenesis. While this lesion can be seen on different imaging modalities, the confirmatory diagnosis relies on the pathologic examination with immunohistochemistry. The majority of GIST shows a benign clinical course, and is intervened with surgical excision. Metastatic potential of this neoplasm is also known, with the liver being the most common organ system to be affected [2,3]. With disseminated disease, a tyrosine kinase inhibitor is rather used. The mechanism of metastasis of GIST or the disease dissemination is not well understood. However, different classifications, such as National Institutes of Health (NIH), Armed Forces Institute of Pathology (AFIP), and modified NIH (mNIH) classifications are used to predict the metastatic potential. The GIST metastasis to the breast is hardly ever discussed. Herein, we report an exceptionally rare case of metastatic GIST in the breast of a 62-year-old female patient, who had had a resection of the primary jejunal GIST. Her disease course was complicated by GIST metastasis to the liver, for which the patient had a living donor liver transplant, and remained on immunosuppressive therapy. Fourteen months after the transplant, she had a breast biopsy proven GIST metastasis. To our knowledge, this is the first reported case of its kind in the United States.

## Case presentation

**Figure 1:**
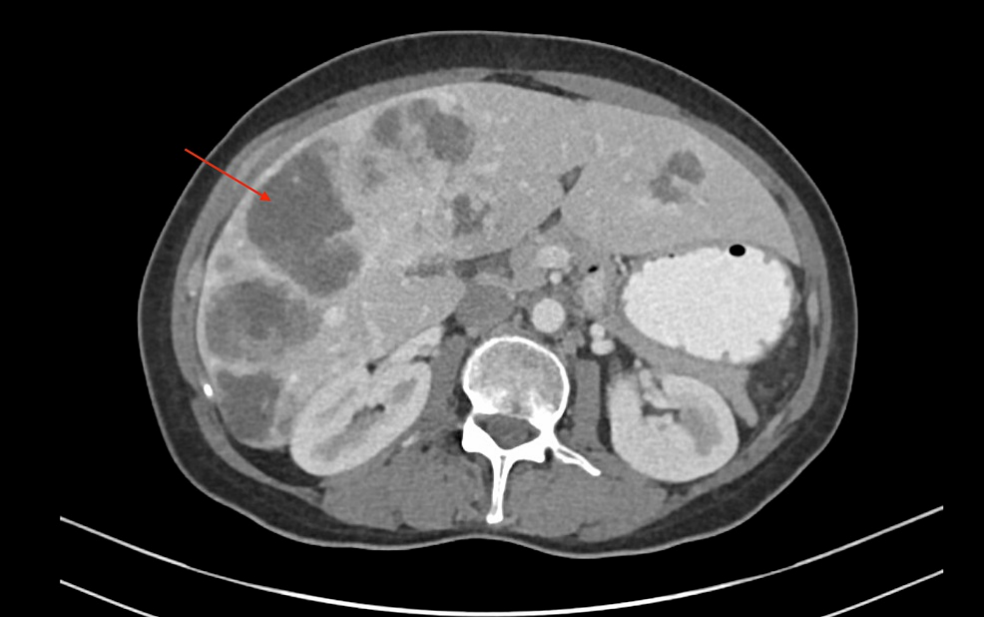
Axial view of computerized tomography imaging. Globally enlarged liver harboring multifocal metastatic GIST, involving both left and right lobes of the liver. GIST, gastrointestinal stromal tumor. Arrow, multifocal metastatic lesions

The patient is a 62-year-old female with a past medical history of asthma, chronic gastritis and with history of GIST of jejunum, who had a resection of the tumor in February 2016, at a different medical institution without adjuvant imatinib therapy. The pathology at the time showed mixed cell type, low-grade GIST (3 mitosis per 5 mm^2^), 3 cm in greatest dimension, with extensive necrosis. All surgical resection margins were negative with the tumor being located more than 1 cm from all margins. In July of the following year, she presented with excessive abdominal pain, and was found to have three hepatic lesions on a surveillance Positron Emission Tomography - Computerized Tomography (PET-CT) scan. The biopsy of the lesions confirmed metastatic GIST. She was then started on imatinib, 400 mg with interval resolution of hypermetabolic activity in the liver without change in size. From January 2018, the patient continued the care at Mount Sinai Health System with stable lesions on surveillance scans, until July 2020, when hepatic lesions showed an interval increase in size (Figure 1).

**Figure 2:**
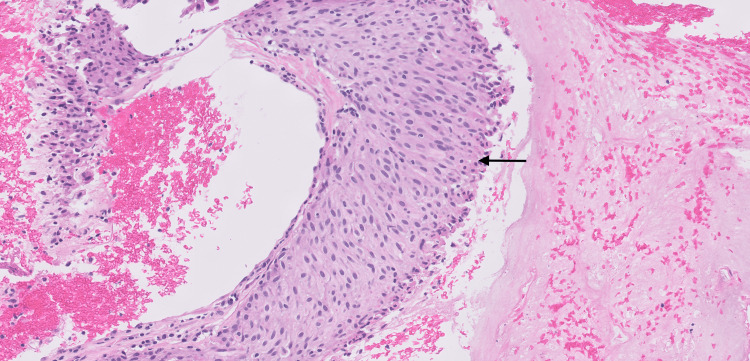
(H&E, x20) Liver biopsy showing predominant epithelioid cell type GIST in the background of fibrins and blood clots. The cellular morphology is blend with nuclei with prominent nucleoli and pink cytoplasm. GIST, gastrointestinal stromal tumor. H&E, hematoxylin and eosin. Arrow, tumor cells

As her previous outside biopsy was not adequate for molecular testing, repeated biopsy of the liver lesion was obtained. On hematoxylin and eosin (H&E) stain, the tumor cells showed epithelioid clusters with relatively bland nuclear morphology (Figure 2).

The neoplastic cells showed diffuse positive immunostaining pattern with c-kit, DOG-1 and rare weak positive with CD34. The specimen was sent for Next-Generation Sequencing (NGS) study, which showed codon 557-558 KIT exon 11 mutation (p.K558_D572del) and KIT exon 17 mutation (p.N822K). An attempt to switch the therapeutic option to sunitinib was aborted due to medication intolerance including nausea, abdominal pain, anorexia, and insomnia. Instead, the patient was put on high dose imatinib, 400 mg twice a day. The latter was also discontinued due to drug-induced hypothyroidism and liver injury in January 2021.

**Figure 3:**
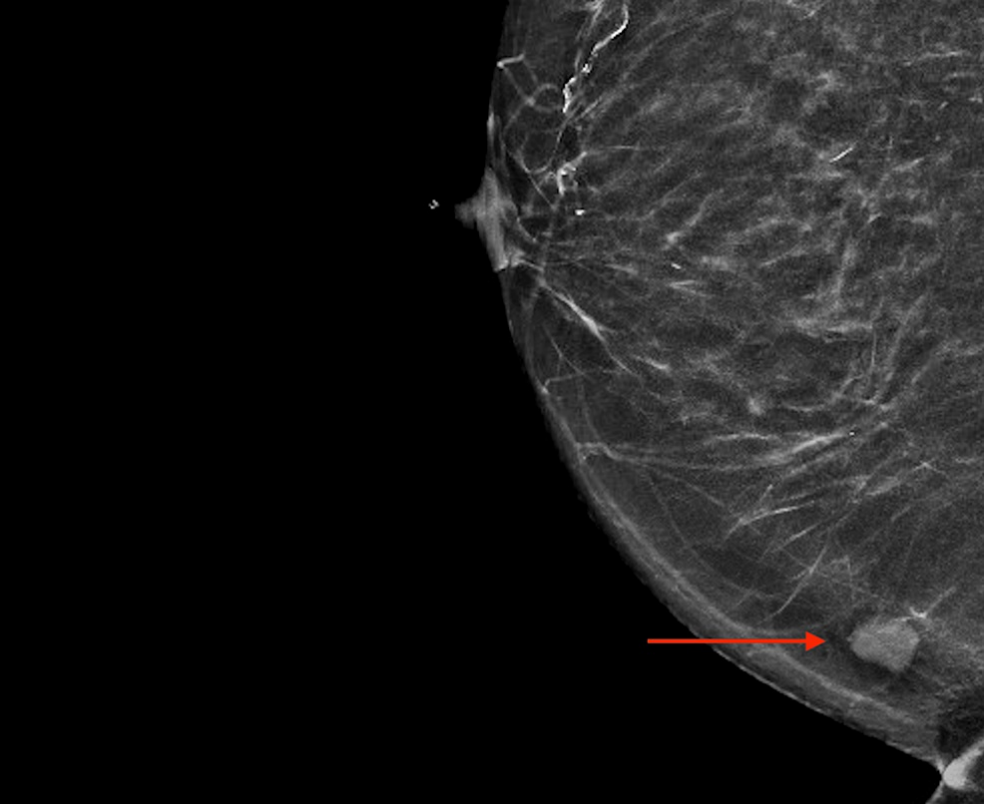
Medio-lateral oblique view of right breast mammography showing ovoid dense well-circumscribed lesion. Arrow, lesion of interest.

Subsequently, with decompensating hepatic function in the setting of the tumor burden isolated to the liver, she had a liver transplant from a living donor relative in March 2021. Intraoperatively, multiple sub-centimeter serosal nodules on the prior site of small bowel anastomosis were identified. The concerned segment was also resected, where the resulting pathology uniformly showed metastatic GIST. With a stable postoperative liver function, the patient was placed on maintenance immunosuppressants, tacrolimus 0.5 mg twice a day and mycophenolate mofetil 500 mg twice a day. The latter was stopped three months post-transplant. Nearly a year later, in early 2022, a follow-up Magnetic Resonance Imaging (MRI) showed interval appearance of new multiple peritoneal deposits and enlarged mesenteric lymph nodes, suspicious for metastasis. In May 2022, 14 months post-transplant, on screening mammographic examination, a circumscribed oval, high density 1.0 cm mass was identified from the right breast on 6:00 axis, 6 cm from the nipple (Figure 3).

**Figure 4:**
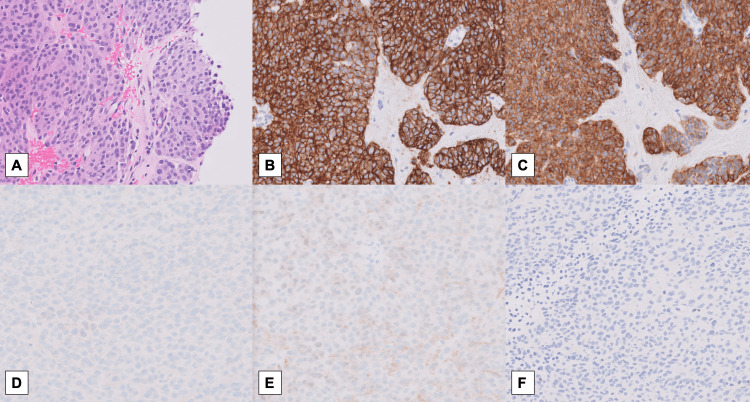
Breast biopsy showing GIST, confirmed by immunohistochemistry. A - Hematoxylin and Eosin slide showing pleomorphic epithelioid cell type GIST with atypical mitoses (H&E, x20). B - Diffuse membranous stain of DOG1 seen in the tumor cells (DOG1, x20). C - Diffuse positive stain of the tumor cell with CD117 (c-Kit, x20). D - Non-reactive ER (ER, x20). E - Non-reactive PR (PR, x20). F - Non-reactive GATA3 (GATA3, x20). H&E, hematoxylin and eosin. GIST, gastrointestinal tumor. DOG1, Discovered On GIST-1. ER, Estrogen receptor. PR, progesterone receptor.

The breast core needle biopsy showed a pleomorphic epithelioid cell-type lesion, with high mitosis (13 mitosis per 5 mm^2^) and extensive necrosis (Figure 4A). The lesion was diffusely positive for DOG1 and CD117 (and patchy weak positive for CD34) and negative for GATA3, ER and PR, confirming the diagnosis of metastatic GIST morphologically and immunohistochemically (Figure 4B-4F).

## Discussion

The incidence of GIST per year is 10 to 15 patients per million, affecting both male and female equally [1]. The disease occurrence is seen across wide age groups, while it is most commonly noted around 60s [1]. GIST is thought to arise from the interstitial cell of Cajal in the GI tract, which functions as a mediator of gastrointestinal peristalsis. This pathological condition can occur anywhere along the gut with the most common primary site being the stomach, followed by the small intestine [4]. At the molecular level, the pathogenesis refers to the mutation of the gene encoding KIT receptor tyrosine kinase in over 80% of the cases or platelet-derived growth receptor alpha (PDGFRA) gene activation in 5%-10% [5]. KIT gene mutation leads to uncontrolled activation of downstream signaling cascade of tyrosine kinase receptor [6]. Among numerous types, exon 11 mutations, as seen in this patient, are most common and associated with a worse prognosis. GIST can also occur without affecting these two genes, in the setting of succinate dehydrogenase complex (SDH) gene mutation, BRAF mutation or, under rare circumstances of syndromic disease such as neurofibromatosis type 1, Carney triad syndrome or Carney-Stratakis syndrome [7,8]. SDH deficient GIST exhibits different disease behaviors than KIT or PDGFRA mutations counterparts, as it can affect patients at younger age, with the female gender predilection, arising only in the stomach, and it can occur multiple in number [9].

The diagnosis of GIST is made from the histopathological characteristics displayed by tumor cells including cellular morphology and immunohistochemistry. Microscopically, the neoplasm can show fusiform looking spindle cell type, epithelioid cell type or mixed cell type, growing in a well-circumscribed whorled pattern architecture. The differential diagnosis of GISTs can include leiomyoma, leiomyosarcoma, and schwannoma. Immunohistochemically, approximately 95% of GISTs stain positive for a transmembrane protein CD117, a tyrosine kinase receptor, which is also inherently positive for some hematopoietic cells, melanocytes and interstitial cells of Cajal [2,10]. Another marker, myeloid progenitor CD34 antigen can be present in the majority of cases, exhibiting the positive staining [10]. S100 is negative, allowing to differentiate this tumor of interest from other mesenchymal neoplasms, such as schwannoma. Moreover, a recently introduced Discovered On GIST-1 (DOG1) antibody marker shows higher sensitivity over the conventional stains, especially in highlighting PDGFRA-derived tumor cells, extra-gastrointestinal stromal tumor (EGIST), and metastatic lesion [11].

The metastatic potential of this neoplasm is uncommon but is a known complication, affecting approximately 10% of the GIST patients [3]. Organs affected by the metastatic disease include liver, bone, lung, and also lymph nodes [2,3]. The latter is rarely affected, except in SDH-deficient GIST patients, where the metastatic involvement of lymphatic tissue is more common [9]. Predicting the metastatic potential is challenging. Few studies have reported different classification systems to assess the malignant potential and the prognostic factors of GIST, such as National Institutes of Health (NIH), Armed Forces Institute of Pathology (AFIP), and modified NIH (mNIH) classifications. These methodologies stratify the malignant potential into four different grades (Table 1).

**Table 1:** GIST grading NIH and mNIH criteria. GIST, Gastrointestinal stromal tumor. NIH, National Institutes of Health. HPF, High Power Field. *: Data reproduced and modified from Fletcher et al.'s study [12]. **: Data reproduced and modified from Huang et al.'s study [13].

NIH* classification			modified NIH** classification		
Risk*	Size*	Mitotic count*	Risk**	Size**	mitotic count**
very low	<2 cm	<5/50 HPF	level I	<2 cm	<5/50 HPF
low	2-5 cm	<5/50 HPF	level II	2-5 cm	6-10/50 HPF
intermediate	<5 cm	6-10/50 HPF		5-10 cm	<5/50 HPF
	5-10 cm	<5/50 HPF	Level III	≤5	>10/50 HPF
high	>5 cm	>5/50 HPF		5-10 cm	6-10/50 HPF
	>10 cm	any mitotic rate		>10	<5/50 HPF
	any size	>10/50 HPF	Level IV	>5	>10/50 HPF

Grading criteria for each classification are based on the size of the tumor and numbers of mitoses [12,13]. In addition, AFIP classification also considers the tumor location (gastric or extra-gastric) [14]. The most favorable outcome is related to tumors arising from gastric location, being smaller than 5 cm in size, and with less than 2 mitoses per 10 high power filed, in the absence of exon 11 KIT mutation [10,14].

Conventionally, GISTs without metastasis are intervened by surgical resection while imatinib (tyrosine kinase inhibitor) is the treatment of choice for metastatic GIST with the majority of patients responding well to the treatment [7,15]. Imatinib acts on PDGFRA and selective tyrosine kinases including abl and c-KIT. Given that most GIST patients have KIT mutation, a positive clinical response to this medication is not surprising. However, unresponsive or imatinib-resistant GISTs may harbor different types of mutation. In addition, as imatinib targets an intracellular domain of ATP-binding site derived from exon 11, and turns off the downstream signaling pathway, any additional mutations affecting other domains may interfere with clinical efficacy of this drug [6]. As for the patients with refractory disease or harboring other than exon 11 KIT mutation, they have alternative therapeutic options such as sunitinib or regorafenib, and avapritinib [15], where sunitinib remains the treatment of choice for refractory disease, irrelevant to the underlying mutation [16]. Different mutations can affect different areas among 21 exons of KIT. Indeed, the presented patient was also found to have a rare exon 17 KIT (p.N822K) mutation, in the setting of exon 11 KIT aberration. Treatment response of such mutation to medical therapy is not well known with few reported cases being refractory to conventional imatinib and/or sunitinib treatments [16].

Spitaleri et al. have pointed out that a minority of non-imatinib-treated GIST patients at the time of primary resection later developed a second mutation affecting exon 17 [16]. However, no potential relationship between the status of imatinib treatment and second mutation has been yet suggested. Another possible association of the aggressive behavior of the neoplasm in the presented patient may be related to the post-transplant immunosuppression.

Immunocompromised status increases the risk of many complications including various infections, metabolic diseases, and even malignancy. Though it is uncertain if the risk of GIST occurrence is also associated, the incidence of this neoplasm in patients on immunosuppressive therapy, such as cyclosporine, azathioprine, prednisone or tacrolimus post-kidney or liver transplant is seldom reported [17,18]. On the same line of thought, the immunosuppressed status on the risk of metastatic behavior of GIST in these patients remained unanswered. Cameron et al. postulated that local immunologic environments may have an influence on the GIST metastasis in a study that included 196 patients [19]. Yet, more data and rigorous study are needed to elucidate the metastatic behavior of GIST to unusual sites, such as breast.

## Conclusions

Gastrointestinal stromal tumor is a mesenchymal neoplasm with a spectrum of presentation. While the majority of the cases show a favorable prognosis, a complicated disease progression, such as metastasis, is a well-known sequela. To our knowledge, a breast metastasis of GIST is yet to be reported in the United States. Spindle cell lesions in the breast most of the time account for metaplastic breast cancer, malignant phyllodes tumor, or rare sarcoma, while GIST is often overlooked as a differential. Consideration of GIST and respective immunochemical work-ups are needed when a clinical suspicion arises.
